# Unveiling the ghost: machine learning’s impact on the landscape of virology

**DOI:** 10.1099/jgv.0.002067

**Published:** 2025-01-13

**Authors:** Sebastian Bowyer, David J. Allen, Nicholas Furnham

**Affiliations:** 1 Department of Infection Biology, Faculty of Infectious and Tropical Diseases, London School of Hygiene and Tropical Medicine, London, UK; 2 Department of Comparative Biomedical Sciences, Section Infection and Immunity, School of Veterinary Medicine, Faculty of Health and Medical Sciences, University of Surrey, Guildford, UK

**Keywords:** deep learning, machine learning, RNA viruses, SARS-CoV-2, virus phenotype prediction, virus evolution

## Abstract

The complexity and speed of evolution in viruses with RNA genomes makes predictive identification of variants with epidemic or pandemic potential challenging. In recent years, machine learning has become an increasingly capable technology for addressing this challenge, as advances in methods and computational power have dramatically improved the performance of models and led to their widespread adoption across industries and disciplines. Nascent applications of machine learning technology to virus research have now expanded, providing new tools for handling large-scale datasets and leading to a reshaping of existing workflows for phenotype prediction, phylogenetic analysis, drug discovery and more. This review explores how machine learning has been applied to and has impacted the study of viruses, before addressing the strengths and limitations of its techniques and finally highlighting the next steps that are needed for the technology to reach its full potential in this challenging and ever-relevant research area.

## Current challenges in virology

Viruses are ubiquitous pathogens which infect virtually all life on the planet. In the context of humans, they are the leading cause of infectious disease and place a major burden on public health worldwide. Viral infections of humans can vary from asymptomatic to life-threatening and, alongside endemic and seasonal illnesses, have the potential to cause major outbreaks and global pandemics. The threat to human health posed by pandemic viruses is exemplified by COVID-19, an infectious disease caused by the coronavirus SARS-CoV-2 that has been responsible for over six million deaths in the 4 years since its emergence in 2019 [1]. Viruses with both high impact on human health and pandemic potential tend to be those with RNA genomes, such as coronaviruses and influenza viruses. The traits of these viruses – typically including high rates of mutation and transmissibility, as well as the ability to infect and jump between the primary animal host and humans as a secondary host – pose a significant and ever-present threat to public health, driven primarily by the unpredictability of a novel virus’ emergence in the human population and the rapid speed of transmission into new communities once it does so [2]. These traits additionally pose a challenge to research efforts and particularly the development of therapeutic strategies, as the speed of evolution and transmission of some emerging viruses with human hosts necessitates a rapid turnaround time and can quickly render antiviral compounds or vaccines ineffective [3]. One avenue for overcoming these challenges is the prediction of new outbreaks through the study of the patterns of mutation and adaptation that give rise to them [4].

We have now entered an era where the number of viral genome sequences available for research is greater than ever before, thanks to the advent of accessible, cost-effective next-generation sequencing (NGS) technology and the unprecedented response of the global health community to COVID-19, with over 16 million SARS-CoV-2 sequences available publicly as of April 2024 [5]. In a similar timeframe, the field of machine learning (ML) has seen an exponential increase in power and capability, driven by the larger volume of data available, technological advancement and the advent of deep learning. Today, ML has already seen focused use as a new avenue for tackling existing challenges in virus research, with many possible applications remaining [6,9].

## An overview of ML

A subfield of artificial intelligence (AI), ML is concerned with the development of mathematical algorithms capable of ‘learning’ from input data – that is, the ability to improve performance on a designated task by analysing new data and without explicit programming. ML algorithms learn the patterns and structure of a training dataset. The output of a trained algorithm is a model, which can then be used to make predictions based on unseen data.

**Fig. 1.**
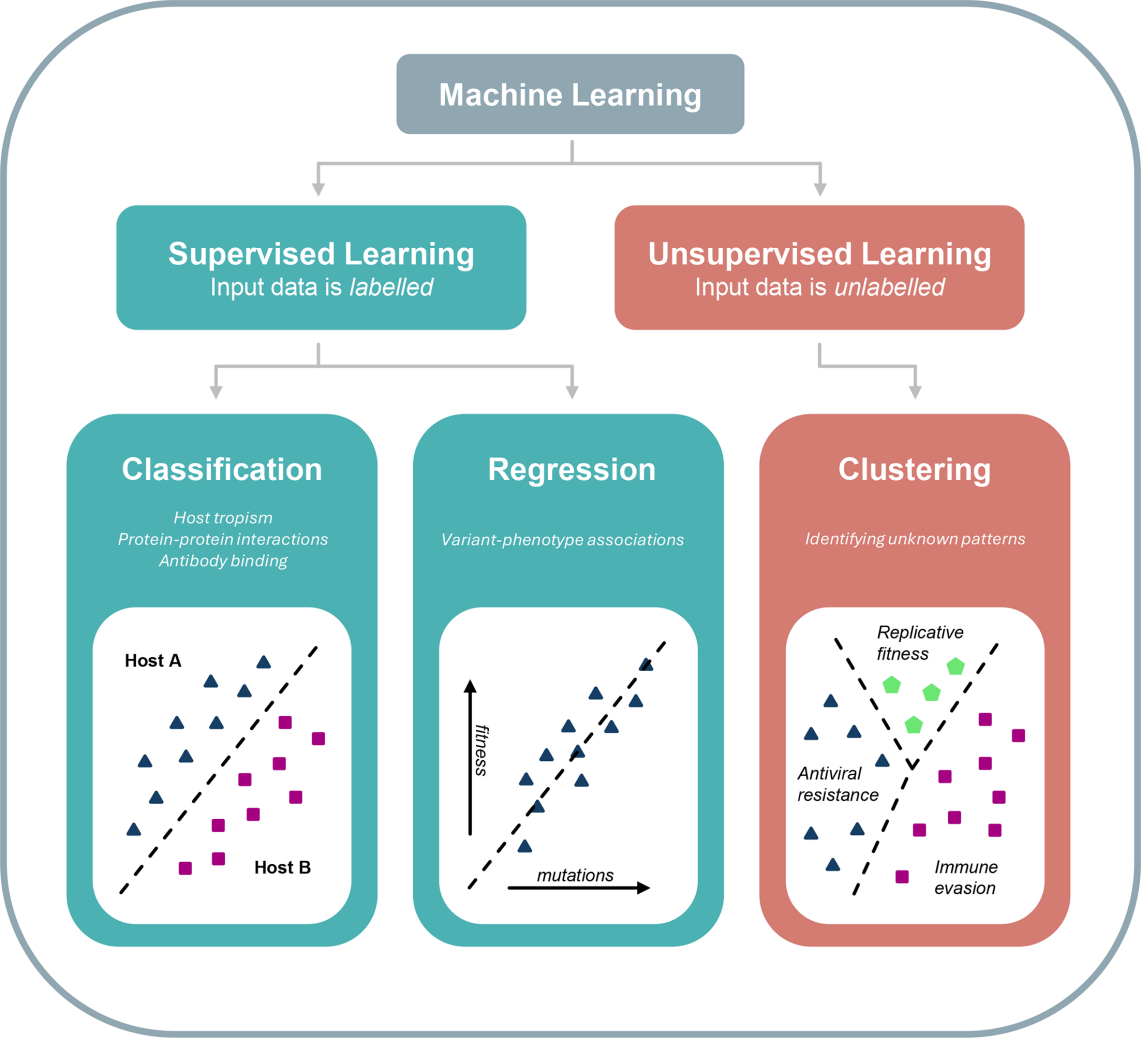
A selection of relevant supervised and unsupervised ML tasks which have been applied to viral data, including representative examples. Classification algorithms place unseen data points into existing categories, based on a labelled training dataset. Classification techniques have seen use for the purposes of predicting tropism from virus genome sequence data, identifying putative interactions between viral and host proteins and predicting whether a given viral protein will bind to a specific antibody [24,95, 103]. Regression algorithms analyse the relationships between dependent and independent variables in data points, with the goal of predicting future unseen values. Regression has been used for the prediction of variant phenotypes [37]. Clustering algorithms group unlabelled data points together based on their similarity to each other. Clustering is used to identify unknown relationships and patterns between data points and phenotypes [89].

A wide array of ML technologies and techniques exist, and each can be broadly categorized based on whether the model is supervised or unsupervised. In supervised learning, training data for an algorithm are labelled in order to teach it a specific goal. In unsupervised learning, the algorithm learns the structure of unlabelled data without explicit instructions. Supervised and unsupervised ML models can then be further defined by the task they perform, which can include the classification of data into categories or the grouping of data based on similarity. These categories and their associated tasks are summarized in Fig. 1. More complex models generally require larger training datasets to perform their tasks effectively. The simplest mathematical models may be able to make accurate predictions based on a training dataset with hundreds or thousands of entries, whereas modern-day language models may require millions in order to identify high-level patterns and nuanced associations between data points [10].

Currently, the latest and most powerful ML models are based on neural networks. A neural network is an algorithm consisting of many interconnected computational nodes. These nodes are also known as neurons, as each will receive, process and output data in a manner similar to those in the human brain [11]. Neural networks have existed conceptually since the mid-twentieth century, though their capabilities have historically been limited due to computational hardware being incapable of hosting complex models with multiple, dense layers of neurons [12]. However, recent technological advances in hardware, network design and training approaches have resulted in the rise of deep learning algorithms – large-scale neural networks with a large number of hidden layers, capable of identifying features and patterns in extremely large and complex datasets. Deep learning algorithms have had a major impact in many areas of research and, in molecular biology, are already known for solving the problem of accurate protein structure prediction from sequence data [13]. Thanks to their rapid advancement and powerful, public-facing applications, deep learning models have come to dominate scientific and cultural discourse in recent years [14].

## ML applied to virus research

**Fig. 2.**
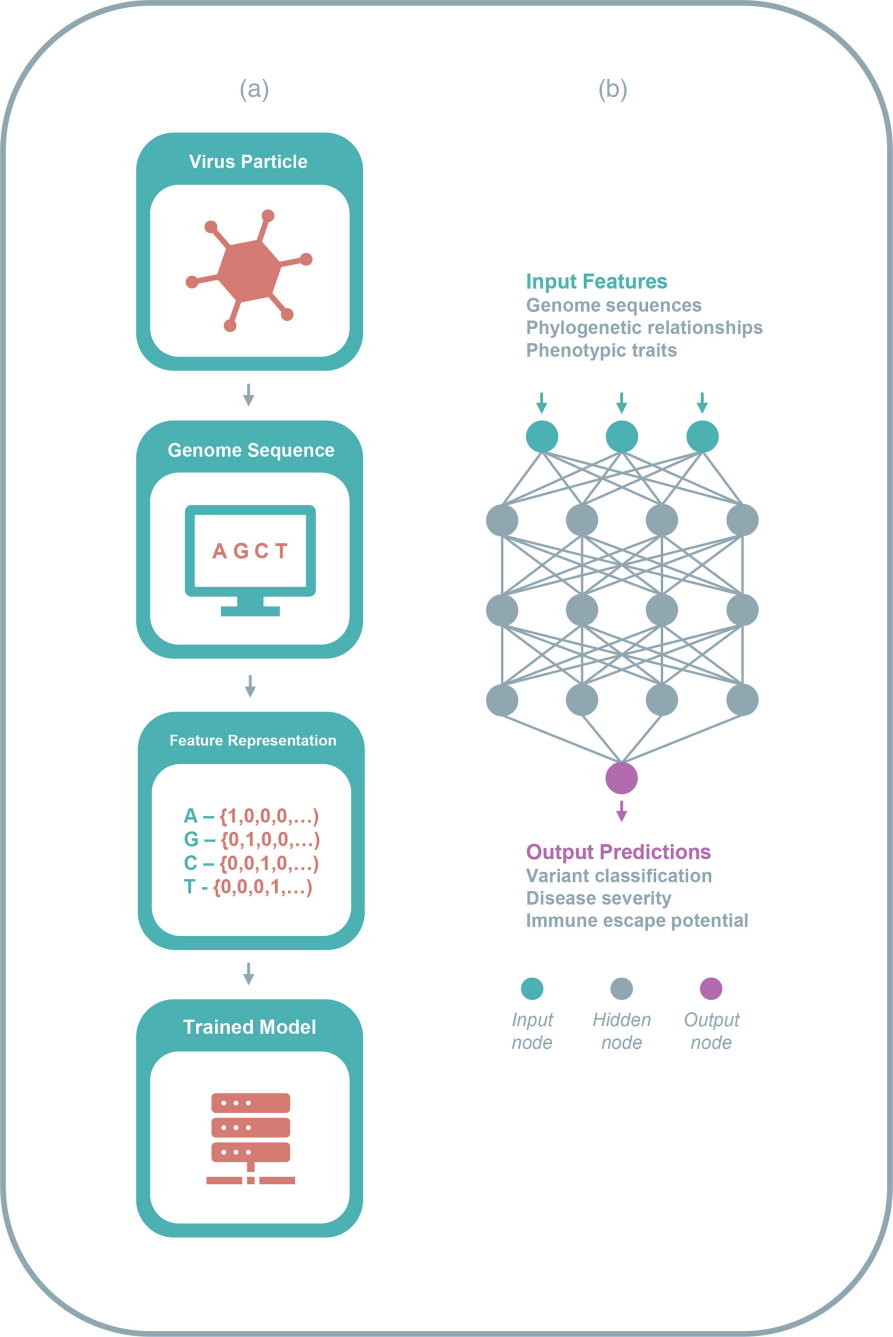
Visualizing ML pathways. (**a)** Preparing and converting viral genomic data for the construction of an ML model. The nucleic acid genome of a virus particle is extracted and sequenced into a form which can be read by computational software. The raw sequence data are then converted into a set of features which can be read by an ML algorithm while maintaining needed information from the original data. The feature set can then be used to train an ML algorithm, ultimately leading to the construction of a completed model. (b) A graphical depiction of a neural network’s structure. Input data are processed by input nodes and passed through a number of hidden layers. Nodes (neurons) receive and process data, before sending it further along the network to other connected nodes. Fully processed data are retrieved from the output node.

Those ML models employed for virus research may work with a wide variety of data types, including structure or binding information, phenotypic or phylogenetic information and nucleic or aa sequences. The latter is frequently utilized and typically encompasses both genome and protein sequences from viruses and their hosts. In order to be used as input for an ML model, data must first be converted into a form that an algorithm can recognize in a process termed feature extraction (Fig. 2). Features are individual measured properties that describe the input data. To provide an example, a feature representation created from an aa sequence may contain a series of values representing its physiochemical properties [15]. There are a wide variety of mathematical techniques for the feature extraction of biological data, with each providing differing representations with varying levels of size and complexity [16]. Feature extraction techniques are chosen based on the composition of the input data, as well as the nature of the ML model being used.

Input data for ML models in virus research are frequently sourced from experimental datasets, the results of simulations or analyses or online databases (Table 1). Many existing studies utilizing ML techniques on viruses take advantage of the large-scale public databases available for genetic sequences, such as GenBank, the BVBRC or GISAID [5,17, 18]. In addition, the focus is shifting increasingly towards the use of general large language models (LLMs) developed from protein structure databases such as the Protein Data Bank or UniProt, for the purposes of advanced feature extraction or addressing sparse training datasets [19,20].

**Table 1.** Available data sources for the training and validation of ML algorithms in virology, broadly categorized with a brief summary of each category as well as use cases and drawbacks for each

Data source	Summary	Use cases	Drawbacks	Ref
**Large-scale, automatically curated databases** (GISAID, NCBI GenBank, Influenza Research Database, Observed Antibody Space, UniProtKB)	Contain hundreds of thousands to millions of sequences and protein structures obtained from around the world; are typically freely available and collaborative efforts, with many groups providing data	Can be used to train or validate a wide variety of ML algorithms, particularly for the purpose of phylogenetic analysis, genotype to phenotype prediction and prediction of protein structure; the extremely large amount of data available can be used to train complex deep learning algorithms, which require comparatively massive training datasets	In many cases, automated curation and the inclusion of entries from many different sources result in overall metadata quality being low, with inconsistencies between entries and very few entries containing phenotypic information, where relevant; overall data may be biassed towards certain regions (particularly Europe and the USA), and in the case of viral sequence databases, data are only available at this scale for heavily studied viruses such as SARS-CoV-2 and influenza	[19,67, 120, 121]
**Large-scale, manually curated databases** (UniProtKB/Swiss-Prot, Protein Database)	Curated datasets containing viral genome sequences or protein structures; manual curation means data must meet strict quality standards, with comprehensive and standardized metadata	Similar to large-scale, automatically curated databases, though the inclusion of additional metadata increases potential ML applications, and high-quality protein structure data can be used for validation	Maintaining and building upon a manually curated, high-quality database requires expert knowledge and significant resources; the Protein Database (PDB) and UniProtKB/Swiss-Prot database are unique examples of such large-scale, manually curated protein structure databases	[122]
**Small-scale, manually curated databases** (SKEMPI, AB Bind)	Curated datasets containing high-quality experimental data collated from existing literature; data vary greatly across datasets and disciplines but may include, for example, thermodynamic and kinetic information for protein–protein interactions (SKEMPI) or antibody binding (AB-Bind)	Used for the training and validation of ML algorithms designed to predict phenotypic effects, such as changes in binding free energy (ΔΔG) due to mutation or relevant thermodynamics	Obtaining and curating experimentally verified data is an extensive process which naturally limits the potential size of these datasets – total entries are typically in the low thousands compared to the millions seen in some genomic databases	[49,123]
**Phenotype databases and ontologies** (Gene Ontology, Database of Genomes and Phenotypes)	Provide information on the functions and phenotypes associated with genes; in the case of ontologies, they aim to develop a standardized vocabulary to describe their effects and interactions and provide evidence-based annotations to relate gene products to their biological role	Can be used to supplement training data for algorithms designed to predict protein–protein interactions, or provide additional information for feature embedding techniques	Consisting of annotations and descriptions, these cannot typically be used as the sole training data for an ML model; less well-known or studied viruses may have fewer or less accurate annotations; likewise, well-known proteins and pathways may have highly complex or detailed annotations and networks, which are difficult to integrate into an ML model	[79,80]
**Derived from experimental data** (NGS, deep mutational scanning, molecular dynamic simulations)	Modern mutagenesis, sequencing and computational techniques allow for novel large-scale datasets to be generated practically by a research laboratory; these datasets can include deep sequencing data on a genetic population, phenotypic data on aa substitutions or simulated data of molecular dynamics and interactions	Used for training and validating algorithms with the goal of phylogenetic analysis or prediction of phenotypic effects. Data may also be used in combination with genomic/structural data to provide phenotypic input for a training dataset	While data from a single experimental source or study may include hundreds of thousands of entries with standardized metadata, this typically lacks the scope of information available from databases with submissions from across the world; additionally, generation of data experimentally requires time, effort and resources in comparison to acquisition from an accessible database; datasets associated with specific studies may not be as readily available as those from collaborative databases	[46,48, 124]

Examples given are not intended to be an exhaustive list – rather representative of each category.

## Classifying viral sequences

One of the more conceptually straightforward tasks in ML is the classification of data into different categories. In the context of virus research, a classification algorithm may be tasked with identifying the host species of a given viral sequence, or assigning it to a genetic lineage. One of the earliest examples of ML being applied to virological data is in the 2009 H1N1 swine flu pandemic, as researchers sought to identify the host origin of newly sequenced viral genomes [21]. Influenza A virus (IAV) is the causative agent of the pandemic and infects a wide range of animal hosts. Additionally, the virus is capable of evading the host immune system either through incremental changes over time or large, dramatic antigenic shift events [22,23]. The ability to quickly determine the origin of IAV genome segments in newly identified sequences enables researchers to more quickly track the emergence, transmission and evolution of influenza viruses.

In a 2009 study, classification algorithms were used to identify a number of IAV sequences obtained from GISAID during the 2009 pandemic as being of either human or swine origin [21]. Each method achieved a prediction accuracy score above 95% for each of the 8 segments in the IAV genome – tempered only by the small training dataset used to create each model (~150 sequences per genome segment, per host) [21]. More recent studies have achieved similar levels of predictive accuracy with much larger training datasets. By representing the aa sequences of each IAV genome segment via their physiochemical properties and applying a random forest classifier, Eng *et al.* achieved 98.62% prediction accuracy when classifying sequences belonging to the IAV fusion protein haemagglutinin (HA) as being of human or avian origin, using a training dataset of ~10 000 sequences [24]. A 2020 study used this foundation to explore feature representation in greater depth, identifying HA as the protein most indicative of host tropism [25]. Lastly, a 2022 study utilizing over 59 000 HA sequences found that a neural network achieved the highest level of accuracy when classifying sequences between human, avian and swine, though at a lower taxonomic level (i.e. specific avian hosts) a gradient-boosting algorithm achieved better results, as these are optimized for dealing with sparse or missing data and the number of sequences available for certain avian hosts is comparatively low [26,27].

Several of the above-mentioned studies have resulted in the construction of online platforms for the prediction of IAV host or subtype from sequence data – using models trained on GISAID or BVBRC (formally IRD) data [18,28, 29]. As a highly impactful pathogen with organized sequencing efforts dating back to the 1980s, IAV has one of the largest sequence datasets of any virus [18]. This puts into perspective the scale of the COVID-19 sequencing effort, with publicly available SARS-CoV-2 sequences already far outnumbering influenza or any other virus. As of November 2024, the GenBank public database contains over 8.9 million SARS-CoV-2 nt sequences (of which 3 million are marked as nt complete) since the start of the pandemic, in comparison to IAV (1.1 million nt sequences, 10 000 complete) and human immunodeficiency virus 1 (HIV-1, 1.1 million nt sequences, 7200 complete), which both number among the most well-represented viruses on the platform with entries dating back as far as 1982 and 1985, respectively [30]. The unprecedented size of the SARS-CoV-2 dataset presents a challenge in how to effectively manage and analyse its entries to gain biological insight – a challenge which ML is well suited to address.

SARS-CoV-2 is a non-segmented, positive-sense RNA virus of zoonotic origin, which replicates efficiently in humans [31]. Due to the proofreading exonuclease capability of the SARS-CoV-2 RNA-dependent RNA polymerase, the virus has a lower replication error rate than IAV (one study has placed the replication error rate for SARS-CoV-2 in cell culture to be 23.9-fold lower than IAV) but has nevertheless accumulated mutations over the course of the COVID-19 pandemic, which has led to the emergence of new variants with altered infectivity and disease phenotypes [32,34]. The ability to quickly classify new SARS-CoV-2 sequences is paramount to tracking the pandemic, and to that end, the Phylogenetic Assignment of Named Global Outbreak lineages (Pangolin or PANGO) nomenclature has been adopted as the standard for the classification of the virus’ genetic lineages [35]. Assigning new viral sequences to a PANGO lineage is performed using the pangoLEARN tool – an ML model consisting of a random forest classification algorithm trained on sequences from the GISAID database, where each base in the SARS-CoV-2 genome has been one-hot encoded [36]. In one-hot encoding, categorical data are converted into a numerical array, which can then be fed to an ML algorithm. In this example, the array represents each of the four nt bases, as well as which of them is present at a given position in the genome. Encoded sequences are then quickly assigned to a lineage.

## Predicting viral phenotypes

Beyond the classification of viral sequences, the ability to predict the relevant phenotypes of novel variants is an important asset for the development of strategies and interventions against existing and emerging viruses. Models built to perform this task may be designed to predict a novel virus’ ability to bind to a specific host receptor, gain resistance to a particular therapy or intervention or evade specific antibodies, among other applications. Such models may also attempt to identify the aa mutations or evolutionary pathways associated with these phenotypes.

PyR_0_ is a logistic regression model trained on over six million SARS-CoV-2 sequences available from GISAID at the time of its development in early 2022 [37]. The goal of the model is to sort sequences into genetic clusters from which it can estimate the overall fitness of a given virus. The PyR_0_ model was constructed using genome sequences with associated collection dates, locations and PANGO lineages – allowing entries to be sorted based on date and region. A complete phylogeny was constructed using a tool specialized for large datasets, and this phylogeny was then used to partition samples into genetic clusters based on a refined version of the PANGO nomenclature [38]. These genetic clusters are used to estimate the fitness of different lineages and form the basis of the model itself, which regresses lineage counts against aa mutations across different times and regions. By aligning differing aa mutations with lineages and fitness estimates across the spatial and temporal history of the pandemic, PyR_0_ demonstrated an ability to detect and characterize new lineages of SARS-CoV-2 (correctly classifying the Omicron variant as having the highest fitness to date at the time of the study) and was able to predict single aa mutations relevant to viral fitness.

However, while powerful, there are limits to PyR_0_ and other regression models when applied to sequence data, as they rely on the assumption that individual mutations are independent of one another in regard to phenotypic effect. In reality, SARS-CoV-2 has repeatedly evolved combinations of mutations, which occur simultaneously, and the evolutionary history of the virus is highly complex and nonlinear to an extent that linear regression algorithms are unable to predict [39,40]. Instead, studies have turned to neural networks and deep learning algorithms to investigate more complex problems.

Working with over seven million spike protein sequences taken from GISAID, the developers of the TEMPO model used a phylogenetic tree-based sampling method to generate historical sequences, which were then fed into a neural network in order to predict aa mutations based on the virus’ evolutionary history [41]. TEMPO was designed for the prediction of potential mutations at target sites based on historical data and successfully predicted 22 out of 39 real-world mutations, which emerged following the most recent entries in its training dataset in February 2022.

Beyond SARS-CoV-2, neural networks have been applied to measure antigenic distances between IAV sequences in both human and swine hosts, allowing for the identification of antigenically distinct strains, which can be selectively targeted for *in vitro* follow-up work [42,43]. In addition, a separate study from Xia *et al.* developed a deep learning model, which employed two separate neural networks – one dealing with local aa context and the other with long-distance sequence information – with the goal of predicting antigenic variation between IAV sequences [44]. This model used a quasi-experimental dataset of phenotypic data from HA inhibition assays and achieved very high prediction performance (99.20% agreement with existing sequence data), though confidence in the model’s performance would be increased with access to a true experimental dataset [45].

Neural networks can also be trained with deep sequencing datasets, which provide high-resolution genetic coverage of their targets [46,47]. Such datasets can include phenotypic information related to specific sequences or mutations. In a recent study, neural networks were applied to data from a deep mutational scanning (DMS) experiment, which leverages deep sequencing alongside laboratory assays to generate phenotypic information for every individual aa substitution mutation in a population [48]. In this case, the DMS experiment investigated the effects of mutations in the receptor-binding domain (RBD) of the SARS-CoV-2 spike protein on binding affinity for the ACE2 receptor, as well as a number of target antibodies. The dataset, which coupled sequence data with phenotypic information, was used to train ML algorithms for the prediction of mutational effects on viral phenotypes, and it was found that a neural network approach was able to most accurately predict binding and non-binding phenotypes – achieving accuracy scores of >0.92, though only when incorporating combinatorial mutations in the training dataset, as well as data that were mutationally distant from the Wu-Hu-1 reference sequence. Due to the multiple nonlinear pathways of SARS-CoV-2 evolution, single aa mutations alone cannot represent the additive effects of combinatorial mutations [49,51].

Phenotypic data obtained from DMS experiments can further be used to supplement ML models trained from sequence or structural information. In a recent study, molecular dynamic simulations, which are capable of analysing the physical movements of atoms and molecules over time in very high resolution, were used to train a neural network model capable of reproducing experimental changes in binding affinity caused by aa mutations in the receptor RBD of the SARS-CoV-2 spike protein [49]. In short, ML analysis performed on snapshots from the simulation was able to estimate the change in binding energy between the RBD and the ACE2 receptor, which could then be directly compared to experimental measurements from a previous DMS experiment in order to validate results. The tool achieved a correlation coefficient of 0.73 between predicted and experimental values. In a separate study, a similar approach revealed interactions in the binding complex between spike and ACE2 [52]. More complex neural networks have proven to be effective tools for the analysis and processing of particularly massive raw datasets outputted by molecular dynamic simulations [53].

## Drug discovery and vaccine design

ML is currently seeing extensive use in pharmaceutical fields as models are applied to pipelines for drug discovery and vaccine design [54,55]. Deep learning is now commonly applied to molecular dynamics and related molecular docking simulations in structure-based drug discovery [56,57]. Briefly, deep learning-based techniques are used to predict protein structures and perform virtual screens, with molecular simulations then being employed to obtain relevant thermodynamic and kinetic data for candidate molecules [56]. An attractive prospect in drug discovery, ML can streamline and focus *in vitro* work to reduce the cost and duration of various steps in the workflow [58].

Deep learning techniques have additionally been employed to perform virtual screenings of potential antiviral compounds and perform functions in molecular dynamic simulations themselves [13,56, 59]. Since the start of the COVID-19 pandemic, deep learning has additionally been used for drug repurposing screenings, identifying a number of existing compounds with activity against SARS-CoV-2 including azathioprine, pralatrexate and, in combination with other compounds, remdesivir [60,63].

In a similar vein, ML techniques are increasingly influencing the related field of vaccine design, via the development of techniques that speed up or replace different stages in the vaccine development pipeline. In particular, ML plays a key role in reverse vaccinology, a long-standing method of vaccine discovery, which identifies potential antigens using computational techniques trained on genetic and immunological data [64].

To date, a number of ML and deep learning-based models have been developed for the prediction of B cell and T cell epitopes as potential vaccine components [65]. Many of these are trained on data from the Immune Epitope Database – a freely available catalogue of experimental data on antibody and T cell epitopes [66]. In addition, deep learning is able to take advantage of advances in NGS-based immune repertoire sequencing for either B cell receptors or T cell receptors – a particularly noteworthy data source for ML algorithms, with some databases containing millions to over a billion sequences [67,68]. Immune repertoire data have been used to train deep learning models focused on sequence classification and prediction of disease severity, though these do represent early applications [69,70].

## Predicting viral host–pathogen interactions

The mechanisms of virus infection and replication are largely determined by protein–protein interactions (PPIs) between the pathogen and host. Understanding these interactions is important for advancing knowledge of virus replication as well as identifying new drug and vaccine targets. As such, the identification of virus–host PPIs has seen extensive research, though the total interactome (complete space of interacting host and viral proteins) remains incomplete, and work continues to be focused on well-studied pathogens [71].

ML methods have seen extensive use for the prediction of PPIs, which can be considered a classification task. PPI classifiers are trained on experimentally validated interacting protein pairs, obtained from host–pathogen PPI databases such as HPIDB, HVIDB or Viruses.STRING for interspecies interactions, or HCVpro and NCBI HIV-1 for specific viruses, for example [72,76]. Interaction data are paired with sequence data for the respective proteins, typically obtained from reviewed structures available from UniProt [19]. As the most abundant form of training data available, aa sequences form the bedrock of most PPI classifiers, with a number of feature extraction techniques employed to discern physiochemical properties, as well as phylogenetic information.

In addition to sequence and interaction data, modern PPI classifiers often incorporate phenotypes associated with specific pathogens or genes [77,78]. These can be acquired from phenotype databases such as PathoPhenoDB or dbGaP, as well as ontology resources, which aim to build and maintain a controlled vocabulary of genes and gene products [79,81]. However, the availability of phenotypic data as well as interaction data can be limited, especially for viruses and hosts, which are less studied [82]. As a result, recent studies have turned to generalizable LLMs to learn more from smaller viral datasets [83].

## Learning the language of viral proteins

LLMs are based on the concepts and techniques of natural language processing. This is a subfield of AI concerned with developing algorithms that can understand, interpret and manipulate human language. Modern natural language processing is based on linguistic techniques and the process of word embedding. In word embedding, individual words are converted to numeric vectors, which can be read by an ML algorithm while preserving semantic context [84,86]. Each of these vectors contains tens to hundreds of dimensions, and in this vector space, any words which are closer to each other are suggested to be similar in meaning. In this way, natural language processing algorithms can take full sequence context into account when performing analysis [87]. While this technology began with the development of tools for speech recognition, translation and language generation, many computational biologists have applied the technology to the language of proteins.

The sequences of aas that make up proteins share much in common with human language regarding their representation, structure and status as ‘information complete’ – i.e. all of a protein’s information is theoretically contained within its aa sequence [88]. As a result, recent studies have attempted to apply natural language processing techniques to the analysis of viral protein sequences, to elucidate or predict the properties of the molecule [10,89].

In one pioneering 2021 study, language models were employed to predict the immune escape potential of protein sequences from IAV, human immunodeficiency virus (HIV) and SARS-CoV-2 [89]. These models were based on neural network architecture and designed to assess grammar (referred to as grammaticality) and semantics as part of the same output. In the context of viral sequences, grammaticality was structured to represent the ‘biological rules’ of infectivity and evolutionary fitness, whereas semantics would refer to a change in the ‘meaning’ of the sequence – in this case the switch to no longer being recognized by the host immune system. The models were able to cluster raw sequence data into groups corresponding to subtype and/or host species, with further analysis indicating that grammatical or semantic change was significantly correlated with fitness and escape potential.

As the field of natural language processing has advanced rapidly in recent years, LLMs have now become the dominant computational tool for ML applications to viral protein data. LLMs are trained on millions or billions of data points and are extremely powerful and versatile, with a vast scale of knowledge and the ability to handle complex tasks and generate fluent and coherent human language [90]. Currently, transformer-based models are at the forefront of natural language processing. These utilize attention mechanisms, where a model can identify and prioritize the most relevant parts of its input sequence. Transformer models form the core of many LLMs, including the BERT and GPT architectures [91]. These have been adapted for use in protein research, with the ProteinBERT model adapting the existing BERT architecture and introducing a pretraining task on over 100 million protein sequences from UniRef90 [10,92]. Similarly, a previously discussed model for the prediction of mutation phenotypes, TEMPO, utilizes transformer architecture to outperform other neural network technologies when predicting mutation effects for SARS-CoV-2 [41,93]. Currently, the evolutionary scale modelling (ESM) family of models are considered among the largest and most powerful protein language models available. These are transformer-based LLMs that can be scaled up to billions of parameters, allowing them to capture high-level evolutionary information and nuanced relationships between sequences in even greater depth. In a recent study, ESM-1b was used in the construction of a new model for the prediction of receptor/antibody binding specificity in SARS-CoV-2 [94]. The study builds upon previous work on natural language applications to viral protein sequences and the prediction of SARS-CoV-2 variant phenotypes to construct ML-guided Antigenic Evolution Prediction (MLAEP), a multitask deep learning model that predicts binding specificity using variant sequences and binding target structures [89,95]. Through experimental validation, it was found that MLAEP was able to predict mutations, which occur in chronic infections as well as emerging variants, and additionally forecast combinatorial mutations through an understanding of epistatic interactions.

The ESM family of models illustrate the ongoing rapid advancement of protein language models. ESM-2, published in 2022, is trained on ~65 million unique protein sequences from UniRef and can hold up to 15 billion parameters [96]. ESM-3, released in 2024, is claimed to hold up to 98 billion parameters [97].

## Addressing data scarcity

With the advent of large protein language models, it is now becoming increasingly common practice to use pre-trained LLMs to obtain input features from sequence data, as generalizable protein models are able to provide a wealth of useful information from a single viral protein sequence. The practice of taking a pre-trained model and adjusting it based on a dataset of interest is called fine-tuning and is a form of transfer learning.

Broadly, transfer learning is concerned with the reapplication of machine knowledge to a new, related task. Its potential lies in its ability to leverage existing models to enable accurate predictions from comparatively sparse information, with a significantly reduced training cost [98]. Within virology, transfer learning has recently been applied to the prediction of PPIs. One recent model, known as multitask transfer, is based on an LLM named UniRep, which is trained on over 24 million sequences from UniRef50 [92,99, 100]. The feature-rich protein representations obtained from this model were then further refined via a neural network, incorporating a multitask component – the prediction of interactions with human proteins. Multitask transfer was found to outperform a number of contemporary models after training on benchmark datasets.

A transfer learning model based on transformer architecture, named DeepVHIPPI, was first pre-trained on the Swiss-Prot database before being fine-tuned with PPI data from SARS-CoV-2, influenza or Ebola [101]. Recently, the STEP architecture, which uses a twin neural network incorporating the pre-trained ProtBERT model, was able to match or beat the accuracy of competing models on the same dataset [102].

## Pitfalls and considerations

While the use of deep learning and fine-tuned protein language models is becoming standard practice for ML applications to viral data, there are still scenarios in which alternative techniques are better suited. In scenarios such as PPI prediction where the ML task can be well defined, smaller-scale algorithms are often more accurate [103,104] and have been employed to predict PPIs for a number of viruses from comparatively small datasets with a high level of accuracy [103,104]. In addition, classical ML techniques are typically less computationally demanding than deep learning, which can require days or weeks of training time with high-performance hardware.

Beyond the specifics of model selection, there are a number of notable considerations that must be made when working with ML models and in the context of the virus research discussed in this review. First, an ML model is only ever as good as the data used to train it – as highlighted in this review, the majority of models trained on viral data have utilized genetic sequences for their training datasets, and such sequences are typically obtained from collaborative online databases. While the wealth of sequencing data available from such platforms are unique resources, which have contributed greatly to research, they are hindered by a lack of associated metadata and a tendency towards regional bias [105]. Metadata elements (such as host tropism, disease severity and therapeutic effectiveness to name a small number of examples) often serve as the predictive goals of trained models but are rarely included in data entries in any significant number (with the notable exception of host tropism). For example, the overwhelming majority of sequences made available on GISAID have no metadata beyond the patient’s age, gender and the date and location of sample collection. As of November 2024, roughly 5% of total sequences in GISAID’s EpiCoV database are listed as having patient status information, and there are a considerable number of entries with missing information. Nevertheless, models like PyR_0_ have shown that SARS-CoV-2 databases can still be used to make accurate phenotypic predictions from viral sequence data and highlight the potential of a dataset that is more robust and richer in metadata. To that end, data scientists are currently pushing initiatives such as FAIR, which call for international standardization of data with robust and comprehensive metadata [106].

In addition, it is important to note that major regional and socioeconomic biases exist within public sequence databases, with the clear majority of samples coming from Europe and the USA [105,107]. While such biases pose clear obstacles to the accurate study of virus diversity and evolution, they also create ethical concerns, as populations from minority or less privileged socioeconomic backgrounds are not fully represented [108,109]. In the related field of cancer biology, population bias has been shown to affect molecular data – a fact that warrants careful consideration and extensive validation when acting from ML predictions [107]. These data disparities highlight the need for a more comprehensive global data infrastructure, as well as open and equal collaboration between collecting institutions.

It can also be seen from this review that the current body of ML research which utilizes viral sequence or protein data is heavily skewed towards SARS-CoV-2, and to a lesser extent IAV. Whilst this is almost certainly due in part to the global importance of the COVID-19 pandemic and its coincidental emergence around the time that deep learning techniques were rapidly advancing, there is also the consideration that SARS-CoV-2 and IAV are two of very few viruses for which sufficient public data are available for the training of ML models. The fact remains that improved data infrastructure is needed for the techniques highlighted in this review to expand beyond this small group of viruses, though transfer learning approaches may hold potential in this regard.

**Fig. 3.**
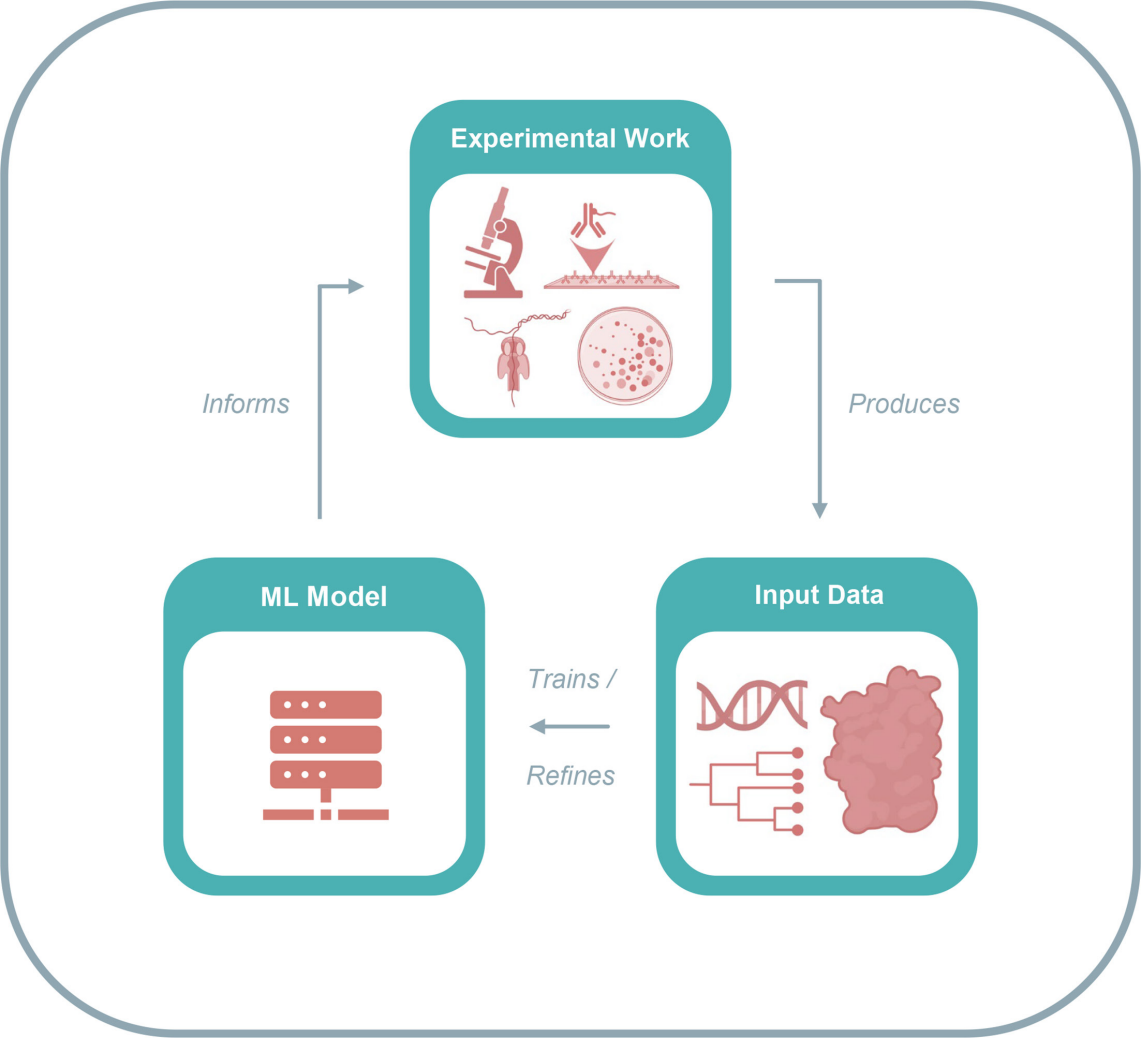
The ‘virtuous cycle’ of the ML workflow. An initial input dataset is used to train an ML model for use on viral data. Model predictions then inform experimental work, which in turn generates additional data used to refine the existing ML model. The refined model then informs further experimental work, and the cycle repeats.

Lastly, it should be considered that deep learning models, including LLMs, can be problematic in scientific research due to their ‘black box’ nature. Due to the self-supervised nature of deep neural network model training and their extreme complexity, it is not possible to understand the mathematical transformations being performed to generate predictions from training data – only to determine the accuracy of the predictions themselves. While efforts are being made to adapt algorithms and construct tools to help us understand the decision-making processes of deep learning through explainable AI, caution should be taken when interpreting ML and deep learning predictions, which should be empirically validated in all cases [110,111]. Whilst many past studies had stopped short of experimental validation of predictions, more recent work has shown that experimental validation of ML predictions can not only serve to improve confidence in model accuracy but also can additionally contribute to a ‘virtuous cycle’, where validated predictions can be fed back into the training dataset to further improve model accuracy and capability (Fig. 3) [94].

## ML in virology – current and future challenges

Some of the earliest applications of ML technology to viral data occurred in the late 2000s and early into the 2010s, where studies utilized ML techniques in an attempt to better understand the 2009 H1N1 swine flu pandemic [21,24]. Since then, the amount and ambition of virology research utilizing ML techniques has increased both in line with ML’s technological development and as the availability of large quantities of data increased. The most recent applications have made use of deep learning models to analyse the unprecedented amount of data generated for SARS-CoV-2 [94]. Looking forwards, it seems clear that protein language models and transfer learning approaches hold the greatest potential for virology, but what advances in virology research might these techniques be capable of enabling?

In order to maximize its potential as part of the virology research toolkit, one major challenge for ML research will be improving generalizability – that is, how effectively a given model can perform across a range of prediction targets. In virology, this idea is particularly important, evidenced by the bias towards SARS-CoV-2 and IAV in ML virus research up to this point. This bias is primarily driven by a lack of sufficient training data for most viruses; however, the advent of LLMs, particularly those trained on generalized protein databases, holds huge potential, as these feature-rich models can be fine-tuned to obtain accurate results from small or sparce datasets. This is evidenced by their increasing use in PPI prediction where studies are frequently limited by data availability – demonstrated with SARS-CoV-2 and human polyomavirus 2 [103].

The ability to analyse and interpret large-scale datasets to generate informed hypotheses is arguably one of the most powerful applications of ML in virology research – once again enabled by large protein language models and their ability to capture the context of the entire protein environment. A powerful application of this approach is in the streamlining of experimental workflows through focusing on research questions.

For example, in drug discovery research, ML approaches offer computational screening of candidate drugs to facilitate the focusing of subsequent *in vitro* studies by conducting *in silico* pre-screening to identify and exclude compounds with unfavourable properties or poor matches and predict those most likely to be successful compounds. This makes the research more cost-effective and has substantially increased the speed at which new compounds can be identified [112]. Furthermore, these ML approaches will be pivotal in advancing the understanding and prediction of evolutionary pathways of viral genomes, particularly in phenotypic prediction of mutations, such as indicating where there may be acquisition of antiviral resistance or immunological escape.

In the case of immune escape, a recent example of an ML model developed to address it is EVEscape – a deep learning model and functional framework for the prediction of immune escape during viral outbreaks (Table 2) [4]. EVEscape is built to predict the likelihood of a given mutation being an escape mutant – able to disrupt polyclonal antibody binding while preserving the other necessary properties of the viral protein. EVEscape is based on EVE, a deep learning model which predicts the pathogenicity of aa variants in humans by analysing evolutionarily related protein sequence alignments from UniRef [113]. As EVE makes predictions based on the likelihood of variants arising in the context of their evolutionary WT, so too is EVEscape able to take epistatic effects into account when making its predictions. EVEscape is designed to be modular and has been trained on a number of small-scale datasets from IAV and SARS-CoV-2, as well as pre-pandemic coronaviruses, HIV and the understudied but high-consequence emerging Lassa and Nipah viruses. With the exception of IAV and HIV, these datasets ranged from ~1000 to ~6000 sequences, and comparison with DMS data confirmed their ability to predict known escape variants. Similarly, the model identified three known escape mutants in Lassa virus within the top 10% of its predictions. The ability of EVEscape to serve as a general framework for predicting viral escape variants makes it a powerful tool for the focusing and prioritization of experimental work in the early stages of an outbreak, as well as contextualizing newly identified virus variants detected by surveillance efforts. In addition, the model has more practical potential owing to its ability to make accurate predictions from comparatively small, pre-pandemic datasets – those with sequence numbers in the low thousands, which is much more representative of publicly available data for many viruses. EVEscape currently runs a publicly accessible web page for the prediction of immune escape for up-to-date SARS-CoV-2 variants on GISAID and flags potentially concerning strains as they emerge [114].

**Table 2.** Tools and models for ML in virus research

Tool	Summary	Access link	Ref
**INFINITy**	A classification tool used to identify the subtype and clade of influenza A and B HA protein sequences; available as an R package or can be accessed via a dedicated web server	infinity.unlu.edu.ar	[28]
**Pangolin**	A command line tool and web application built for the rapid assignment of genetic lineages to SARS-CoV-2 sequences; lineage assignment is performed via pangoLEARN, an ML classification algorithm	pangolin.cog-uk.io	[36]
**EVEscape**	A tool which predicts the likelihood of antibody escape for mutations across a number of different viruses; based on EVE, a deep learning model trained on evolutionarily related protein sequences	evescape.org	[4]
**UniRep**	A deep learning model which provides feature-rich protein representation for downstream engineering task; trained on over 24 million protein sequences	github.com/churchlab/UniRep	[100]
**ProteinBERT**	A transformer-based LLM based on the BERT architecture and trained on over 100 million protein sequences; a highly generalizable model which can be fine-tuned for any protein-related task	github.com/nadavbra/protein_bert	[10]
**ESM3**	The latest in the ESM series of LLMs, trained as a general model of proteins; ESM3 is trained on 2.78 billion protein sequences and can be used to generate new, functional protein sequences	evolutionaryscale.ai	[97]

Generalizable and modular tools like EVEscape may offer a glimpse into the future of ML models trained on viral sequence and protein data, and these models may play a key role in addressing both present and future virus research and public health challenges. Where good surveillance systems exist, such as for IAV, predictive ML tools may be able to infer virus evolutionary trajectories and identify variants with concerning mutations before their transmission becomes established in the population, giving an opportunity to prepare countermeasures such as response infrastructure and updated vaccine formulations. When outbreaks do occur, generalizable models may be able to identify drug or vaccine targets and forecast their efficacy, allowing a focused response effort to be deployed much more quickly.

Climate change is altering – and will continue to alter – the ecology and epidemiology of infectious diseases, seen already by changing patterns of vector-borne viruses, including West Nile, Zika and dengue [115]. Already, ML techniques have been used to develop dengue outbreak prediction models and integrated approaches to analyse arbovirus epidemiology alongside geospatial mapping of their respective vectors [116,118]. As the global climate crisis continues, many vector species of viral infections will expand into non-endemic areas, changing disease burden and risk populations. The use of ML has potential in underpinning research, and supporting public health responses to ensure the global community is prepared for future outbreaks.

While this review has been chiefly concerned with ML applications to viral sequence and protein data, it is acknowledged that ML technology has also seen extensive application to clinical data. In this area, ML models have been developed for disease diagnosis, treatment and prognosis among many other applications, and while considered beyond the scope of this review, it is likely that clinical ML research will intersect with molecular ML research in the future [119].

Fully realizing the potential for ML to transform approaches for future virology research and response will require international cooperation and investment. This must be done in order to improve data infrastructure, ensure data collected conform to established standards, expand surveillance programmes co-ordinately with training and infrastructure equitably across all resource settings and geographies and enable open, unrestricted access to viral sequence and protein datasets. Further, these approaches must be implemented for both established and emerging viruses.

## Abbreviations

AI: artificial intelligence
DMS: deep mutational scanning
ESM: evolutionary scale modelling
HA: haemagglutinin
HIV: human immunodeficiency virus
HIV-1: human immunodeficiency virus 1
IAV: influenza A virus
LLMs: large language models
ML: machine learning
MLAEP: ML-guided antigenic evolution prediction
NGS: next-generation sequencing
PPI: protein–protein interaction
RBD: receptor-binding domain

## References

[1] World Health Organisation WHO COVID-19 Dashboard. [accessed December 2023]. https://covid19.who.int.

[2] Pike BL, Saylors KE, Fair JN, Lebreton M, Tamoufe U (2010). The origin and prevention of pandemics. Clin Infect Dis.

[3] Liu C, Hu L, Dong G, Zhang Y, Ferreira da Silva-Júnior E (2023). Emerging drug design strategies in anti-influenza drug discovery. Acta Pharm Sin B.

[4] Thadani NN, Gurev S, Notin P, Youssef N, Rollins NJ (2023). Learning from prepandemic data to forecast viral escape. Nature.

[5] Elbe S, Buckland-Merrett G (2017). Data, disease and diplomacy: GISAID’s innovative contribution to global health. *Glob Chall*.

[6] Tarca AL, Carey VJ, Chen X, Romero R, Drăghici S (2007). Machine learning and its applications to biology. PLoS Comput Biol.

[7] Goodswen SJ, Barratt JLN, Kennedy PJ, Kaufer A, Calarco L (2021). Machine learning and applications in microbiology. FEMS Microbiol Rev.

[8] Ching T, Himmelstein DS, Beaulieu-Jones BK, Kalinin AA, Do BT (2018). Opportunities and obstacles for deep learning in biology and medicine. J R Soc Interface.

[9] Zhang Y, Ye T, Xi H, Juhas M, Li J (2021). Deep learning driven drug discovery: tackling severe acute respiratory syndrome coronavirus 2. Front Microbiol.

[10] Brandes N, Ofer D, Peleg Y, Rappoport N, Linial M (2022). ProteinBERT: a universal deep-learning model of protein sequence and function. Bioinformatics.

[11] Zou J, Han Y, So SS, Livingstone DJ (2009). Artificial Neural Networks: Methods and Applications [Internet].

[12] Macukow B, Saeed K, Homenda W (2016). Computer Information Systems and Industrial Management.

[13] Jumper J, Evans R, Pritzel A, Green T, Figurnov M (2021). Highly accurate protein structure prediction with AlphaFold. Nature.

[14] Zhao WX, Zhou K, Li J, Tang T, Wang X (2023). A Survey of Large Language Models [Internet]. arXiv. http://arxiv.org/abs/2303.18223.

[15] Govindan G, Nair AS (2013). Bagging with CTD-a novel signature for the hierarchical prediction of secreted protein trafficking in eukaryotes. Genom Proteom Bioinform.

[16] Detlefsen NS, Hauberg S, Boomsma W (2022). Learning meaningful representations of protein sequences. Nat Commun.

[17] Sayers EW, Bolton EE, Brister JR, Canese K, Chan J (2022). Database resources of the national center for biotechnology information. Nucleic Acids Res.

[18] Olson RD, Assaf R, Brettin T, Conrad N, Cucinell C (2023). Introducing the Bacterial and Viral Bioinformatics Resource Center (BV-BRC): a resource combining PATRIC, IRD and ViPR. Nucleic Acids Res.

[19] Bateman A, Martin M-J, Orchard S, Magrane M, Ahmad S (2023). UniProt: the universal protein knowledgebase in 2023. Nucleic Acids Res.

[20] Berman HM, Westbrook J, Feng Z, Gilliland G, Bhat TN (2000). The protein data bank. Nucleic Acids Res.

[21] Attaluri PK, Zheng X, Chen Z, Lu G (2009). Applying machine learning techniques to classify H1N1 viral strains occurring in 2009 flu pandemic. BIOT-2009.

[22] Kim H, Webster RG, Webby RJ (2018). Influenza virus: dealing with a drifting and shifting pathogen. Viral Immunol.

[23] Webster RG, Bean WJ, Gorman OT, Chambers TM, Kawaoka Y (1992). Evolution and ecology of influenza A viruses. Microbiol Rev.

[24] Eng CLP, Tong JC, Tan TW (2014). Predicting host tropism of influenza A virus proteins using random forest. BMC Med Genom.

[25] Kwon E, Cho M, Kim H, Son HS (2020). A study on host tropism determinants of influenza virus using machine learning. Curr Bioinform.

[26] Xu Y, Wojtczak D (2022). Dive into machine learning algorithms for influenza virus host prediction with hemagglutinin sequences. Biosystems.

[27] Chen T, Guestrin C (2016). XGBoost: a scalable tree boosting system.

[28] Cacciabue M, Marcone DN (2023). INFINITy: a fast machine learning-based application for human influenza A and B virus subtyping. Influenza Other Respir Viruses.

[29] Humayun F, Khan F, Fawad N, Shamas S, Fazal S (2021). Computational method for classification of avian influenza A virus using DNA sequence information and physicochemical properties. Front Genet.

[30] (2023). NCBI Virus [Internet]. https://www.ncbi.nlm.nih.gov/labs/virus/vssi.

[31] Hui KPY, Ho JCW, Cheung M-C, Ng K-C, Ching RHH (2022). SARS-CoV-2 Omicron variant replication in human bronchus and lung ex vivo. Nature.

[32] Robson F, Khan KS, Le TK, Paris C, Demirbag S (2020). Coronavirus RNA proofreading: molecular basis and therapeutic targeting. Mol Cell.

[33] Carabelli AM, Peacock TP, Thorne LG, Harvey WT, Hughes J (2023). SARS-CoV-2 variant biology: immune escape, transmission and fitness. Nat Rev Microbiol.

[34] Kawasaki Y, Abe H, Yasuda J (2023). Comparison of genome replication fidelity between SARS-CoV-2 and influenza A virus in cell culture. Sci Rep.

[35] Rambaut A, Holmes EC, O’Toole Á, Hill V, McCrone JT (2020). A dynamic nomenclature proposal for SARS-CoV-2 lineages to assist genomic epidemiology. Nat Microbiol.

[36] O’Toole Á, Scher E, Underwood A, Jackson B, Hill V (2021). Assignment of epidemiological lineages in an emerging pandemic using the pangolin tool. Virus Evol.

[37] Obermeyer F, Jankowiak M, Barkas N, Schaffner SF, Pyle JD (2022). Analysis of 6.4 million SARS-CoV-2 genomes identifies mutations associated with fitness. Science.

[38] Turakhia Y, Thornlow B, Hinrichs AS, De Maio N, Gozashti L (2021). Ultrafast Sample placement on Existing tRees (UShER) enables real-time phylogenetics for the SARS-CoV-2 pandemic. Nat Genet.

[39] Lucas C, Vogels CBF, Yildirim I, Rothman JE, Lu P (2021). Impact of circulating SARS-CoV-2 variants on mRNA vaccine-induced immunity. Nature.

[40] Baj A, Novazzi F, Drago Ferrante F, Genoni A, Tettamanzi E (2021). Spike protein evolution in the SARS-CoV-2 delta variant of concern: a case series from Northern Lombardy. Emerg Microbes Infect.

[41] Zhou B, Zhou H, Zhang X, Xu X, Chai Y (2023). TEMPO: a transformer-based mutation prediction framework for SARS-CoV-2 evolution. Comput Biol Med.

[42] Zeller MA, Gauger PC, Arendsee ZW, Souza CK, Vincent AL (2021). Machine learning prediction and experimental validation of antigenic drift in H3 influenza A viruses in Swine. mSphere.

[43] Yao Y, Li X, Liao B, Huang L, He P (2017). Predicting influenza antigenicity from *Hemagglutintin* sequence data based on a joint random forest method. Sci Rep.

[44] Xia YL, Li W, Li Y, Ji XL, Fu YX (2021). A deep learning approach for predicting antigenic variation of influenza A H3N2. Comput Math Methods Med.

[45] Smith DJ, Lapedes AS, de Jong JC, Bestebroer TM, Rimmelzwaan GF (2004). Mapping the antigenic and genetic evolution of influenza virus. Science.

[46] Goldman D, Domschke K (2014). Making sense of deep sequencing. Int J Neuropsychopharmacol.

[47] Schmidt B, Hildebrandt A (2021). Deep learning in next-generation sequencing. Drug Discov Today.

[48] Fowler DM, Fields S (2014). Deep mutational scanning: a new style of protein science. Nat Methods.

[49] Chen C, Boorla VS, Banerjee D, Chowdhury R, Cavener VS (2021). Computational prediction of the effect of amino acid changes on the binding affinity between SARS-CoV-2 spike RBD and human ACE2. Proc Natl Acad Sci USA.

[50] Starr TN, Greaney AJ, Hannon WW, Loes AN, Hauser K (2022). Shifting mutational constraints in the SARS-CoV-2 receptor-binding domain during viral evolution. Science.

[51] Markov PV, Ghafari M, Beer M, Lythgoe K, Simmonds P (2023). The evolution of SARS-CoV-2. Nat Rev Microbiol.

[52] Pavlova A, Zhang Z, Acharya A, Lynch DL, Pang YT (2021). Machine learning reveals the critical interactions for SARS-CoV-2 spike protein binding to ACE2. J Phys Chem Lett.

[53] Casalino L, Dommer AC, Gaieb Z, Barros EP, Sztain T (2021). AI-driven multiscale simulations illuminate mechanisms of SARS-CoV-2 spike dynamics. Int J High Perform Comput Appl.

[54] Thomas S, Abraham A, Baldwin J, Piplani S, Petrovsky N (2022). Artificial intelligence in vaccine and drug design. Methods Mol Biol.

[55] Dara S, Dhamercherla S, Jadav SS, Babu CM, Ahsan MJ (2022). Machine learning in drug discovery: a review. Artif Intell Rev.

[56] Sun Y, Jiao Y, Shi C, Zhang Y (2022). Deep learning-based molecular dynamics simulation for structure-based drug design against SARS-CoV-2. Comput Struct Biotechnol J.

[57] Joshi T, Sharma P, Mathpal S, Joshi T, Maiti P (2022). Computational investigation of drug bank compounds against 3C-like protease (3CL^pro^) of SARS-CoV-2 using deep learning and molecular dynamics simulation. Mol Divers.

[58] Ozdemir ES, Ranganathan SV, Nussinov R (2022). How has artificial intelligence impacted COVID-19 drug repurposing and what lessons have we learned?. Expert Opin Drug Discov.

[59] Liu Z, Du J, Fang J, Yin Y, Xu G (2019). DeepScreening: a deep learning-based screening web server for accelerating drug discovery. Database.

[60] Beck BR, Shin B, Choi Y, Park S, Kang K (2020). Predicting commercially available antiviral drugs that may act on the novel coronavirus (SARS-CoV-2) through a drug-target interaction deep learning model. Comput Struct Biotechnol J.

[61] Zhang H, Yang Y, Li J, Wang M, Saravanan KM (2020). A novel virtual screening procedure identifies pralatrexate as inhibitor of SARS-CoV-2 RdRp and it reduces viral replication *in vitro*. PLoS Comput Biol.

[62] Beigel JH, Tomashek KM, Dodd LE (2020). Remdesivir for the treatment of covid-19 - preliminary report. reply. N Engl J Med.

[63] Ali M, Park IH, Kim J, Kim G, Oh J (2023). How deep learning in antiviral molecular profiling identified anti-SARS-CoV-2 inhibitors. Biomedicines.

[64] Ponne S, Kumar R, Vanmathi SM, Brilhante RSN, Kumar CR (2024). Reverse engineering protection: a comprehensive survey of reverse vaccinology-based vaccines targeting viral pathogens. Vaccine.

[65] Bukhari SNH, Jain A, Haq E, Mehbodniya A, Webber J (2022). Machine learning techniques for the prediction of B-Cell and T-Cell epitopes as potential vaccine targets with a specific focus on SARS-CoV-2 pathogen: a review. Pathogens.

[66] Vita R, Overton JA, Greenbaum JA, Ponomarenko J, Clark JD (2015). The immune epitope database (IEDB) 3.0. Nucleic Acids Res.

[67] Olsen TH, Boyles F, Deane CM (2022). Observed Antibody Space: a diverse database of cleaned, annotated, and translated unpaired and paired antibody sequences. Protein Sci.

[68] Guo Y, Chen K, Kwong PD, Shapiro L, Sheng Z (2019). cAb-Rep: a database of curated antibody repertoires for exploring antibody diversity and predicting antibody prevalence. Front Immunol.

[69] Sidhom JW, Larman HB, Pardoll DM, Baras AS (2021). DeepTCR is a deep learning framework for revealing sequence concepts within T-cell repertoires. Nat Commun.

[70] Schultheiß C, Paschold L, Simnica D, Mohme M, Willscher E (2020). Next-generation sequencing of T and B cell receptor repertoires from COVID-19 patients showed signatures associated with severity of disease. Immunity.

[71] Lian X, Yang X, Yang S, Zhang Z (2021). Current status and future perspectives of computational studies on human–virus protein–protein interactions. Brief Bioinform.

[72] Ammari MG, Gresham CR, McCarthy FM, Nanduri B (2016). HPIDB 2.0: a curated database for host-pathogen interactions. Database.

[73] Yang X, Lian X, Fu C, Wuchty S, Yang S (2021). HVIDB: a comprehensive database for human-virus protein-protein interactions. *Brief Bioinform*.

[74] Cook HV, Doncheva NT, Szklarczyk D, von Mering C, Jensen LJ (2018). Viruses.STRING: a virus-host protein-protein interaction database. Viruses.

[75] Kwofie SK, Schaefer U, Sundararajan VS, Bajic VB, Christoffels A (2011). HCVpro: hepatitis C virus protein interaction database. Infect Genet Evol.

[76] Ako-Adjei D, Fu W, Wallin C, Katz KS, Song G (2015). HIV-1, human interaction database: current status and new features. Nucleic Acids Res.

[77] Liu-Wei W, Kafkas Ş, Chen J, Dimonaco NJ, Tegnér J (2021). DeepViral: prediction of novel virus-host interactions from protein sequences and infectious disease phenotypes. Bioinformatics.

[78] Tastan O, Qi Y, Carbonell JG, Klein-seetharaman J (2008). Prediction of interactions between HIV-1 and human proteins by information integration. Biocomputing.

[79] Ashburner M, Ball CA, Blake JA, Botstein D, Butler H (2000). Gene ontology: tool for the unification of biology. Nat Genet.

[80] Tryka KA, Hao L, Sturcke A, Jin Y, Wang ZY (2014). NCBI’s database of genotypes and phenotypes: dbGaP. Nucleic Acids Res.

[81] Kafkas Ş, Abdelhakim M, Hashish Y, Kulmanov M, Abdellatif M (2019). PathoPhenoDB, linking human pathogens to their phenotypes in support of infectious disease research. Sci Data.

[82] Brito AF, Pinney JW (2017). Protein-protein interactions in virus-host systems. Front Microbiol.

[83] Yang X, Yang S, Lian X, Wuchty S, Zhang Z (2021). Transfer learning via multi-scale convolutional neural layers for human-virus protein-protein interaction prediction. Bioinformatics.

[84] Tsukiyama S, Hasan MM, Fujii S, Kurata H (2021). LSTM-PHV: prediction of human-virus protein-protein interactions by LSTM with word2vec. *Brief Bioinform*.

[85] Yang X, Yang S, Li Q, Wuchty S, Zhang Z (2020). Prediction of human-virus protein-protein interactions through a sequence embedding-based machine learning method. Comput Struct Biotechnol J.

[86] Ibtehaz N, Kihara D (2021). Application of Sequence Embedding in Protein Sequence-Based Predictions [Internet]. arXiv. http://arxiv.org/abs/2110.07609.

[87] Yao Y, Du X, Diao Y, Zhu H (2019). An integration of deep learning with feature embedding for protein–protein interaction prediction. PeerJ.

[88] Ofer D, Brandes N, Linial M (2021). The language of proteins: NLP, machine learning & protein sequences. Comput Struct Biotechnol J.

[89] Hie B, Zhong ED, Berger B, Bryson B (2021). Learning the language of viral evolution and escape. Science.

[90] Patil R, Gudivada V (2024). A review of current trends, techniques, and challenges in Large Language Models (LLMs). Appl Sci.

[91] Devlin J, Chang MW, Lee K, Toutanova K. B (2019). Pre-training of deep bidirectional transformers for language understanding. https://aclanthology.org/N19-1423.

[92] Suzek BE, Huang H, McGarvey P, Mazumder R, Wu CH (2007). UniRef: comprehensive and non-redundant UniProt reference clusters. Bioinformatics.

[93] Asgari E, Mofrad MRK (2015). Continuous distributed representation of biological sequences for deep proteomics and genomics. PLoS One.

[94] Han W, Chen N, Xu X, Sahil A, Zhou J (2023). Predicting the antigenic evolution of SARS-COV-2 with deep learning. Nat Commun.

[95] Taft JM, Weber CR, Gao B, Ehling RA, Han J (2022). Deep mutational learning predicts ACE2 binding and antibody escape to combinatorial mutations in the SARS-CoV-2 receptor-binding domain. Cell.

[96] Lin Z, Akin H, Rao R, Hie B, Zhu Z (2023). Evolutionary-scale prediction of atomic-level protein structure with a language model. Science.

[97] Hayes T, Rao R, Akin H, Sofroniew NJ, Oktay D (2024). Simulating 500 million years of evolution with a language model. Synth Biol.

[98] Iman M, Arabnia HR, Rasheed K (2023). A review of deep transfer learning and recent advancements. Technologies.

[99] Dong TN, Brogden G, Gerold G, Khosla M (2021). A multitask transfer learning framework for the prediction of virus-human protein-protein interactions. BMC Bioinform.

[100] Alley EC, Khimulya G, Biswas S, AlQuraishi M, Church GM (2019). Unified rational protein engineering with sequence-based deep representation learning. Nat Methods.

[101] Lanchantin J, Weingarten T, Sekhon A, Miller C, Qi Y (2021). Proceedings of the 12th ACM Conference on Bioinformatics, Computational Biology, and Health Informatics [Internet].

[102] Madan S, Demina V, Stapf M, Ernst O, Fröhlich H (2022). Accurate prediction of virus-host protein-protein interactions via a siamese neural network using deep protein sequence embeddings. Patterns.

[103] Cui G, Fang C, Han K (2012). Prediction of protein-protein interactions between viruses and human by an SVM model. BMC Bioinf.

[104] Karabulut OC, Karpuzcu BA, Türk E, Ibrahim AH, Süzek BE (2021). ML-AdVInfect: a machine-learning based adenoviral infection predictor. Front Mol Biosci.

[105] Chen Z, Azman AS, Chen X, Zou J, Tian Y (2022). Global landscape of SARS-CoV-2 genomic surveillance and data sharing. Nat Genet.

[106] Wilkinson MD, Dumontier M, IjJ A, Appleton G, Axton M (2016). The FAIR guiding principles for scientific data management and stewardship. Sci Data.

[107] Spratt DE, Chan T, Waldron L, Speers C, Feng FY (2016). Racial/ethnic disparities in genomic sequencing. JAMA Oncol.

[108] Juhn YJ, Ryu E, Wi CI, King KS, Malik M (2022). Assessing socioeconomic bias in machine learning algorithms in health care: a case study of the HOUSES index. J Am Med Inform Assoc.

[109] Abbud A, Castilho EA (2021). A call for a more comprehensive SARS-cov-2 sequence database for Brazil. Lancet Reg Health Am.

[110] Linardatos P, Papastefanopoulos V, Kotsiantis S (2021). Explainable AI: a review of machine learning interpretability methods. Entropy.

[111] Dasari CM, Bhukya R (2022). Explainable deep neural networks for novel viral genome prediction. *Appl Intell*.

[112] Askr H, Elgeldawi E, Aboul Ella H, Elshaier YAMM, Gomaa MM (2023). Deep learning in drug discovery: an integrative review and future challenges. Artif Intell Rev.

[113] Frazer J, Notin P, Dias M, Gomez A, Min JK (2021). Disease variant prediction with deep generative models of evolutionary data. Nature.

[114] Thanadi N, Gurev S, Notin P, Youssef N, Rollins N (2023). Learning from pre-pandemic data to forecast viral escape. [accessed May 2024]. https://evescape.org/.

[115] Paz S (2024). Climate change: a driver of increasing vector-borne disease transmission in non-endemic areas. PLoS Med.

[116] Leung XY, Islam RM, Adhami M, Ilic D, McDonald L (2023). A systematic review of dengue outbreak prediction models: current scenario and future directions. PLoS Negl Trop Dis.

[117] Alexander J, Wilke ABB, Mantero A, Vasquez C, Petrie W (2022). Using machine learning to understand microgeographic determinants of the Zika vector, *Aedes aegypti*. PLoS One.

[118] Rahman MS, Chamsai P, Sumaira Z, Tipaya E, Richard EP (2022). Mapping the spatial distribution of the dengue vector *Aedes aegypti* and predicting its abundance in northeastern Thailand using machine learning approach. One health.

[119] Mano LY, Torres AM, Morales AG, Cruz CCP, Cardoso FH (2023). Machine learning applied to COVID-19: a review of the initial pandemic period. Int J Comput Intell Syst.

[120] Khare S, Gurry C, Freitas L, Schultz MB, Bach G (2021). GISAID’s role in pandemic response. China CDC Wkly.

[121] Bao Y, Bolotov P, Dernovoy D, Kiryutin B, Zaslavsky L (2008). The influenza virus resource at the national center for biotechnology information. J Virol.

[122] Poux S, Arighi CN, Magrane M, Bateman A, Wei C-H (2017). On expert curation and scalability: UniProtKB/swiss-prot as a case study. Bioinformatics.

[123] Jankauskaite J, Jiménez-García B, Dapkunas J, Fernández-Recio J, Moal IH (2019). SKEMPI 2.0: an updated benchmark of changes in protein-protein binding energy, kinetics and thermodynamics upon mutation. Bioinformatics.

[124] Hospital A, Goñi JR, Orozco M, Gelpí JL (2015). Molecular dynamics simulations: advances and applications. *Adv Appl Bioinform Chem*.

